# Clinical Performance of Contrast-Enhanced Mammography Versus Breast MRI in Women at Increased Breast Cancer Risk

**DOI:** 10.3390/cancers18050759

**Published:** 2026-02-27

**Authors:** Gisella Gennaro, Enrica Baldan, Iliana Bednarova, Paolo Belli, Daniela Bernardi, Elisabetta Bezzon, Giovanna Ciriello, Alessandro Coran, Valentina Iotti, Ilaria Polico, Stefania Zovato, Francesca Caumo

**Affiliations:** 1 Breast Radiology Unit, Veneto Institute of Oncology (IOV), IRCCS, 35128 Padua, Italy; enrica.baldan@iov.veneto.it (E.B.); iliana.bednarova@iov.veneto.it (I.B.); elisabetta.bezzon@iov.veneto.it (E.B.); alessandro.coran@iov.veneto.it (A.C.); ilaria.polico@iov.veneto.it (I.P.); francesca.caumo@iov.veneto.it (F.C.); 2 Department of Radiological and Haematological Sciences—Section of Radiology, Università Cattolica del Sacro Cuore, 00168 Rome, Italy; paolo.belli@policlinicogemelli.it; 3 Department of Diagnostic Imaging and Oncological Radiotherapy, Fondazione Policlinico Universitario Agostino Gemelli IRCCS, 00168 Rome, Italy; 4 Humanitas Clinical and Research Center-IRCCS, Humanitas Cancer Center, 20089 Rozzano, Italy; daniela.bernardi@hunimed.eu; 5 Department of Biomedical Sciences, Humanitas University, 20072 Pieve Emanuele, Italy; 6 Istituto Dermopatico dell’Immacolata (IDI)–IRCCS, 00167 Rome, Italy; g.ciriello@idi.it; 7 Radiology Unit, Department of Diagnostic Imaging and Laboratory Medicine, Azienda USL, IRCCS di Reggio Emilia, 42123 Reggio Emilia, Italy; valentina.iotti@ausl.re.it; 8 Hereditary Tumor Unit, Veneto Institute of Oncology (IOV), IRCCS, 35128 Padua, Italy; stefania.zovato@iov.veneto.it

**Keywords:** mammography, magnetic resonance imaging, contrast media, breast cancer, high-risk populations

## Abstract

**Background/Objectives**: Surveillance of women at increased risk for breast cancer requires high-sensitivity imaging. This study compared contrast-enhanced mammography (CEM) with low-energy CEM (LE-CEM) and breast MRI in this population. **Methods**: This retrospective analysis included 461 women enrolled in a high-risk imaging protocol (March 2019–October 2022). Lifetime breast cancer risk was estimated using the Tyrer–Cuzick model. All participants underwent CEM and breast MRI ≥ 72 h apart. LE-CEM images were used as a surrogate for digital mammography. Four readers independently interpreted LE-CEM and CEM; a separate group of four readers interpreted MRI. Diagnostic performance was assessed using sensitivity, specificity, and area under the receiver operating characteristic curve (AUC) within a multireader, multicase framework. Noninferiority testing was performed with Δ = 0.05. Mean glandular dose (MGD) for CEM was recorded. **Results**: CEM showed higher sensitivity than LE-CEM (83.0% vs. 68.9%, *p* < 0.001) with similar specificity (90.8% vs. 92.5%, *p* = 0.176). Mean AUC increased from 0.856 for LE-CEM to 0.936 for CEM (*p* < 0.001). CEM and MRI showed comparable sensitivity (82.9% vs. 86.1%, *p* = 0.547), specificity (90.8% vs. 86.5%, *p* = 0.318), and mean AUC (0.936 vs. 0.933; *p* = 0.839), confirming noninferiority of CEM. MGD per view ranged from 1.56 to 3.10 mGy. **Conclusions**: Contrast-enhanced mammography provides diagnostic accuracy equivalent to breast MRI and superior to LE-CEM, with acceptable radiation dose, supporting its use for surveillance in women at increased breast cancer risk.

## Simple Summary

Women at increased risk for breast cancer need highly sensitive imaging to detect tumors early. Breast MRI is effective but can be costly, time-consuming, or unavailable in some settings. Contrast-enhanced mammography (CEM) is a newer imaging method that combines standard mammography with a safe contrast agent to highlight areas of abnormal blood flow in the breast. In this study, we compared CEM with low-energy CEM (used as a surrogate of mammography) and breast MRI in 461 high-risk women. We found that CEM detected more cancers than low-energy images and performed as well as breast MRI, while maintaining a safe radiation dose. These results suggest that CEM could be a practical, reliable, and widely accessible option for monitoring women at increased risk of breast cancer, potentially improving early detection and guiding personalized screening strategies.

## 1. Introduction

Breast cancer screening strategies for women at increased risk remain an area of active investigation. Current international guidelines, including those from the American Cancer Society and the European Society of Breast Imaging, recommend annual breast MRI in addition to mammography for women at high risk, with adjustments based on age and individual risk profile. MRI alone is typically performed in very young women due to the limited sensitivity of mammography in dense breasts, whereas in older women annual mammography may be proposed when MRI is not feasible [1,2,3,4,5]. Although this combined approach maximizes early detection, it is resource-intensive and constrained by several limitations. MRI requires specialized equipment, dedicated expertise, and intravenous gadolinium administration, and is contraindicated in patients with severe renal impairment, certain implants, or claustrophobia [6]. High cost and limited availability further restrict access, especially in screening of high-risk women [7,8].

Contrast-enhanced mammography (CEM) has emerged as a promising adjunct or alternative in this setting [9,10]. CEM combines standard digital mammography with iodine-based functional imaging, enhancing lesion conspicuity through visualization of tumor vascularity similar to dynamic contrast-enhanced MRI [11]. The low-energy (LE) images acquired during CEM are equivalent to digital mammography in terms of image quality and breast density, despite the presence of contrast agent in the breast [12,13]. This equivalence allows radiologists to interpret LE-CEM as a conventional mammogram, combining morphological information with the functional information provided by the contrast-enhanced image. Moreover, since the initial studies on CEM, it has been recommended that a separate standard mammography is not required in addition to CEM, as this would increase radiation dose without additional clinical benefit [14,15]. This approach enables direct comparison within a single CEM examination, avoiding an extra mammographic acquisition. Multiple studies have shown that CEM offers substantially higher sensitivity than mammography alone and approaches the diagnostic performance of MRI, with early evidence of improved specificity [16,17]. Large multicenter trials, including ECOG-ACRIN EA1141 and BRAID, have further supported CEM as an accessible and cost-effective alternative for cancer detection, particularly in women with dense breasts or elevated risk [18,19]. Importantly, modern dual-energy CEM protocols achieve mean glandular doses within established international safety limits and comparable to digital breast tomosynthesis [20,21].

Growing interest in the use of CEM for surveillance of women at increased risk reflects its potential to provide broader access to functional breast imaging while maintaining high diagnostic performance [22]. However, direct head-to-head comparisons between CEM and MRI in women at increased risk remain limited. Only a few studies have evaluated these modalities using rigorous multireader, multicase methodologies, and evidence stratified by clinically relevant factors such as individual risk level and breast density is scarce [23,24,25].

To address this gap, we conducted a study comparing the diagnostic performance of CEM and breast MRI in women at intermediate and high risk, while also evaluating low-energy CEM as a surrogate for mammography.

## 2. Materials and Methods

### 2.1. Study Design and Population

This study was a secondary, retrospective analysis of a prospectively enrolled cohort of women at increased risk for breast cancer. The protocol was approved by the Institutional Ethics Committee (CE IOV #2017/92), and all participants provided written informed consent.

Between March 2019 and October 2022, 490 women aged ≥35 years at increased risk for breast cancer were prospectively enrolled in a dedicated high-risk imaging protocol and underwent both CEM and breast MRI. Individual lifetime risk was estimated using the Tyrer–Cuzick breast cancer risk model (IBIS v.8) [26,27], with classification according to the NICE guidelines: intermediate risk (17–30%) and high risk (>30%) [28]. Mean volumetric breast density, used as a risk factor for lifetime risk estimation, was measured using Volpara v.2.03 [27,29]. Women with known pathogenic germline mutations (e.g., BRCA1, BRCA2, PALB2) were included [30].

CEM and MRI were performed at least 72 h apart to avoid interference between contrast agents.

### 2.2. Imaging Protocols

*Contrast-Enhanced Mammography*

All CEM examinations were performed on a GE Senographe Pristina system (GE Healthcare, Chicago, IL, USA) in automatic exposure mode. Iodinated contrast (iohexol, Omnipaque 350; GE Healthcare) was administered at 1.5 mL/kg, 2 min before acquisition. Bilateral cranio-caudal (CC) and medio-lateral oblique (MLO) views were obtained.

LE images were used as surrogate for standard digital mammography, and high-energy (HE) images were processed via dual-energy subtraction (DES) to generate contrast-enhanced images.

*Magnetic Resonance Imaging*

MRI was performed on a 1.5-T system (Magnetom Essenza/Expree; Siemens Healthineers, Erlangen, Germany) with a dedicated bilateral breast coil. The protocol included dynamic contrast-enhanced T1-weighted 3D gradient-echo sequences acquired before and after intravenous gadobutrol (0.1 mmol/kg), with five post-contrast phases at ~60 s intervals. Additional sequences included T2-weighted and diffusion-weighted imaging for tissue characterization. Subtraction and maximum intensity projection (MIP) reconstructions were generated for review. All studies were performed prone with bilateral coverage.

### 2.3. Retrospective Reading Protocol

A multireader, multicase (MRMC) analysis was conducted retrospectively using anonymized datasets. Two reader groups (each comprising four breast radiologists) participated, all with prior experience with the respective modality to minimize learning-curve effects:

- Group 1 (R1–R4): experienced in CEM (2–5 years of experience, F.C., V.I., I.B., D.B.).
- Group 2 (R5–R8): breast MRI experts (8–15 years of experience, E.Ba., P.B., A.C., I.P.).

LE-CEM and DES images were reviewed sequentially by R1–R4 on a dedicated workstation (SenoIris, GE Healthcare). MRI studies were independently evaluated by R5–R8 using a standard DICOM viewer (MicroDicom, MicroDicom Ltd., Sofia, Bulgaria). Readers were blinded to clinical information, histopathology, and other modality results. Reading sessions were divided to reduce fatigue.

For LE-CEM, up to three findings per case were recorded: breast density, lesion location, size, type, conspicuity, and BI-RADS score. For CEM and MRI, BPE, lesion location, size, type (up to three per case), conspicuity, and BI-RADS score were recorded.

### 2.4. MRMC Analysis and Statistical Methods

Two complementary multireader, multicase (MRMC) designs were applied. A factorial design was used for the comparison between LE-CEM and CEM, in which the same readers (R1–R4) interpreted the same cases under both conditions, allowing within-reader comparisons. A nested design was used for the comparison between CEM and MRI, with the same cases interpreted by two distinct reader groups (R1–R4 vs. R5–R8), accounting for the hierarchical reader–case structure. Variance analysis was adapted for each comparison to reflect the study design.

The ground truth was histopathology for lesions that underwent biopsy or surgery. Lesions not subjected to biopsy were classified as benign if they remained stable or resolved on imaging follow-up of at least 24 months. Negative cases were similarly verified through a minimum of 24 months of imaging follow-up.

The primary outcome was diagnostic performance between modalities: (1) mammography (LE-CEM) vs. CEM, and (2) CEM vs. MRI, assessed using receiver operating characteristic (ROC) analysis within the MRMC framework. Secondary outcomes were sensitivity and specificity. For these calculations, a BI-RADS score > 3 (i.e., 4–5) was considered positive for malignancy, while scores ≤ 3 were considered negative, in line with standard clinical practice.

Analyses were performed overall and stratified by breast cancer risk (intermediate vs. high), breast density (low vs. high, i.e., a + b vs. c + d), and menopausal status (pre-/peri- vs. post-menopausal).

AUC was estimated for each reader and modality. Treatment effects were analyzed using mean ROC curve and analysis of variance (ANOVA) with a multireader, multicase framework, implemented with the R package MRMCaov v. 0.3.0 (“Multi-Reader Multi-Case Analysis of Variance”, January 2025) in RStudio 2025.09.2. Noninferiority analyses were performed for AUC, sensitivity, and specificity with a predefined margin of Δ = 0.05, which was chosen based on prior breast imaging studies as a clinically acceptable threshold for equivalence in diagnostic performance between modalities. A two-sided *p* < 0.05 was considered statistically significant.

Finally, mean glandular dose (MGD) per view for CEM was recorded and analyzed according to compressed breast thickness interval and compared with EU reference limits for digital mammography and tomosynthesis.

## 3. Results

### 3.1. Study Population

**Figure 1:**
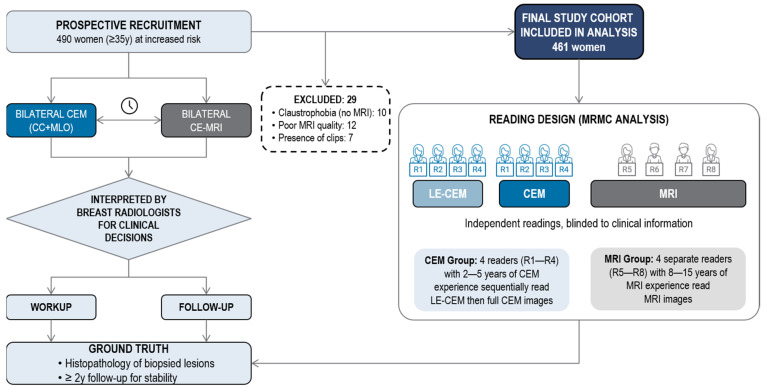
Flow diagram of study population. Of the 490 women prospectively enrolled in the high-risk screening program, 29 were excluded due to MRI refusal for claustrophobia (n = 10), suboptimal MRI quality (n = 12), or the presence of metallic clips affecting lesion visibility (n = 7), resulting in a final study cohort of 461 women included in the analysis. CC = cranio-caudal, CEM = contrast-enhanced mammography, DM = digital mammography, FU = follow-up, HR = high risk, IR = intermediate risk, LE = low-energy, LTR = lifetime risk, MLO = medio-lateral oblique, MRI = magnetic resonance imaging.

Of the 490 participants, 29 were excluded due to claustrophobia preventing MRI acquisition (n = 10), suboptimal MRI quality (n = 12), or the presence of metallic clips affecting lesion visibility (n = 7), leaving 461 women for analysis (Figure 1).

The mean age was 49.8 ± 9.0 years, with a mean BMI of 23.8 ± 4.4 kg/m^2^. More than half were premenopausal (52.5%), with 10.0% perimenopausal and 37.5% post-menopausal. Risk stratification identified 135 women (29.3%) at intermediate risk and 326 (70.7%) at high risk, including 48 BRCA1, 63 BRCA2, and 2 PALB2 mutation carriers. Imaging was performed for surveillance in 342 women (74.2%) and for diagnostic assessment in 119 (25.8%). Overall, 119 cancers (25.8%) were detected, with 16 (9.1%) in the surveillance cohort and 103 (76.9%) in the assessment cohort (Table 1).

### 3.2. Diagnostic Performance of LE-CEM, CEM, and MRI

**Figure 2:**
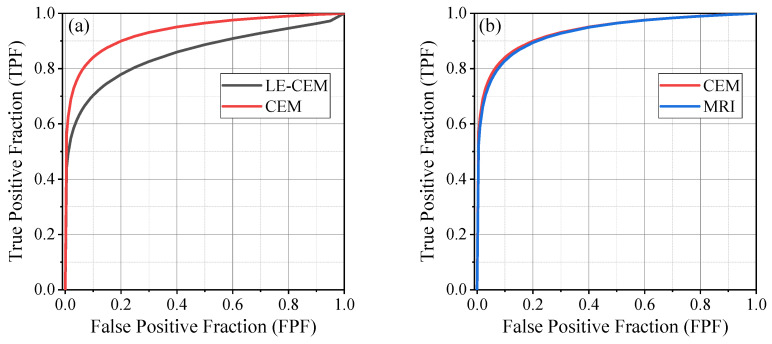
Receiver operating characteristic (ROC) curves illustrating diagnostic performance for (**a**) low-energy contrast-enhanced mammography (LE-CEM, used as a surrogate for digital mammography) versus contrast-enhanced mammography (CEM), and (**b**) CEM versus breast MRI.

Across the four CEM readers, contrast-enhanced mammography (CEM) consistently demonstrated higher sensitivity than low-energy CEM (LE-CEM, used as a surrogate for digital mammography), while specificity remained comparable. Sensitivity ranged from 57.1% to 75.6% for LE-CEM and 73.9% to 89.1% for CEM, whereas specificity ranged from 87.1% to 97.7% for LE-CEM and 87.7% to 95.3% for CEM. Area under the ROC curve (AUC) improved for all readers with CEM (0.924–0.953) compared with LE-CEM (0.821–0.897). The mean AUC increased from 0.856 (95% CI, 0.803–0.909) for LE-CEM to 0.936 (95% CI, 0.911–0.962) for CEM, representing a statistically significant improvement in diagnostic performance (*p* < 0.001, Table 2, Figure 2a).

When comparing CEM with MRI, mean AUC values were similar (0.936 vs. 0.933; *p* = 0.839), with overlapping sensitivity (73.9–89.1% vs. 80.7–89.9%) and specificity (87.7–95.3% vs. 78.9–92.1%) ranges across readers, indicating comparable overall diagnostic performance (Table 2, Figure 2b).

The confusion matrices for each reader, showing true-positive (TP), false-positive (FP), true-negative (TN), and false-negative (FN) counts, are reported in Appendix A.1 and Appendix A.2 (Table A1 and Table A2).

### 3.3. Diagnostic Performance Stratified by Risk, Breast Density, and Menopausal Status

Table 3 provides the diagnostic performance of LE-CEM, CEM, and MRI averaged across readers, with 95% confidence intervals, stratified by risk (intermediate vs. high), breast density (a + b vs. c + d), and menopausal status (pre/perimenopausal vs. post-menopausal).

In intermediate-risk women, mean AUC increased from 0.880 (LE-CEM) to 0.956 (CEM), with significant sensitivity improvement (77.7% to 89.8%, *p* < 0.001) and a nonsignificant reduction in specificity (89.1% to 85.1%, *p* = 0.100). CEM and MRI were equivalent in this subgroup (AUC 0.956 vs. 0.957; *p* > 0.6). Among high-risk women, CEM outperformed LE-CEM, increasing mean AUC from 0.775 to 0.903 (*p* = 0.029) and sensitivity from 58.0% to 74.5% (*p* = 0.002), while specificity remained stable. Compared with MRI, CEM showed similar AUC (0.903 vs. 0.899), sensitivity, and specificity (*p* > 0.2).

In low-density breasts (a + b), CEM increased AUC from 0.860 to 0.930 (*p* = 0.148) and sensitivity from 60.3% to 75.0% (*p* = 0.010), while specificity remained stable. CEM and MRI demonstrated comparable AUC (0.930 vs. 0.927; *p* = 0.94) and sensitivity/specificity, confirming equivalent diagnostic performance. High-density breasts (c + d) showed a statistically significant improvement of CEM over LE-CEM (AUC 0.936 vs. 0.840; *p* < 0.001), with increased sensitivity (70.3% to 84.3%; *p* < 0.001) and stable specificity. CEM and MRI were equivalent in this subgroup (AUC 0.936 vs. 0.932; *p* = 0.65).

Pre-/peri-menopausal women demonstrated increased AUC and sensitivity with CEM compared with LE-CEM (AUC 0.927 vs. 0.809; sensitivity 82.8% vs. 65.3%), with similar specificity. In post-menopausal women, CEM also improved AUC (0.950 vs. 0.882) and sensitivity (74.0% vs. 57.0%), without significant specificity changes.

### 3.4. Noninferiority Analysis

The primary noninferiority endpoint was AUC, with a prespecified margin of −0.05. Overall, CEM was noninferior to MRI, with a mean AUC difference of +0.004 (95% CI −0.034 to 0.041; *p* = 0.839) (Table 4). Subgroup analyses revealed that noninferiority was achieved in intermediate-risk women (−0.009, 95% CI −0.032 to 0.030), high-density breasts (+0.004, 95% CI −0.035 to 0.044), and pre-/peri-menopausal women (+0.021, 95% CI −0.037 to 0.079).

**Figure 3:**
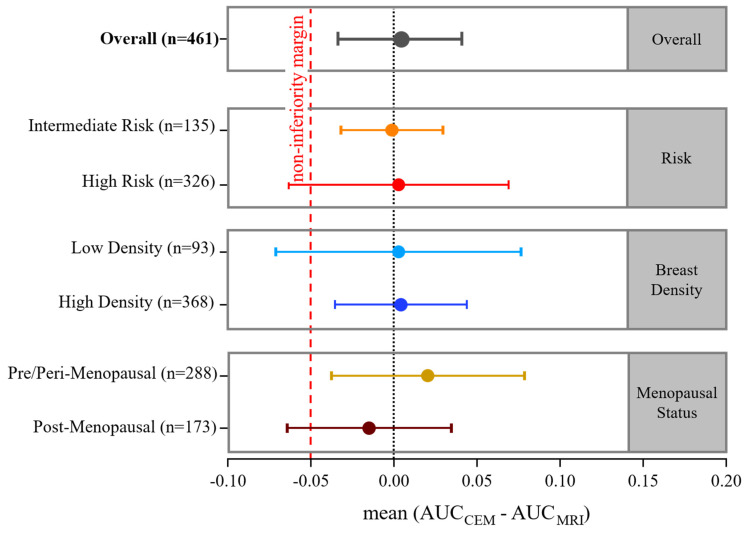
Forest plot illustrating mean AUC difference between CEM and MRI and 95% confidence intervals, overall and stratified by risk, breast density, and menopausal status.

In contrast, high-risk women (+0.003, 95% CI −0.063 to 0.069), low-density breasts (+0.003, 95% CI −0.071 to 0.077), and post-menopausal women (−0.015, 95% CI −0.064 to 0.035) had lower confidence bounds slightly below the noninferiority margin, formally not meeting the criterion. Nonetheless, the absolute differences were minimal and centered near zero, as illustrated in Figure 3.

Data showing CEM superiority over LE-CEM are presented in Appendix A, Table A3.

**Figure 4:**
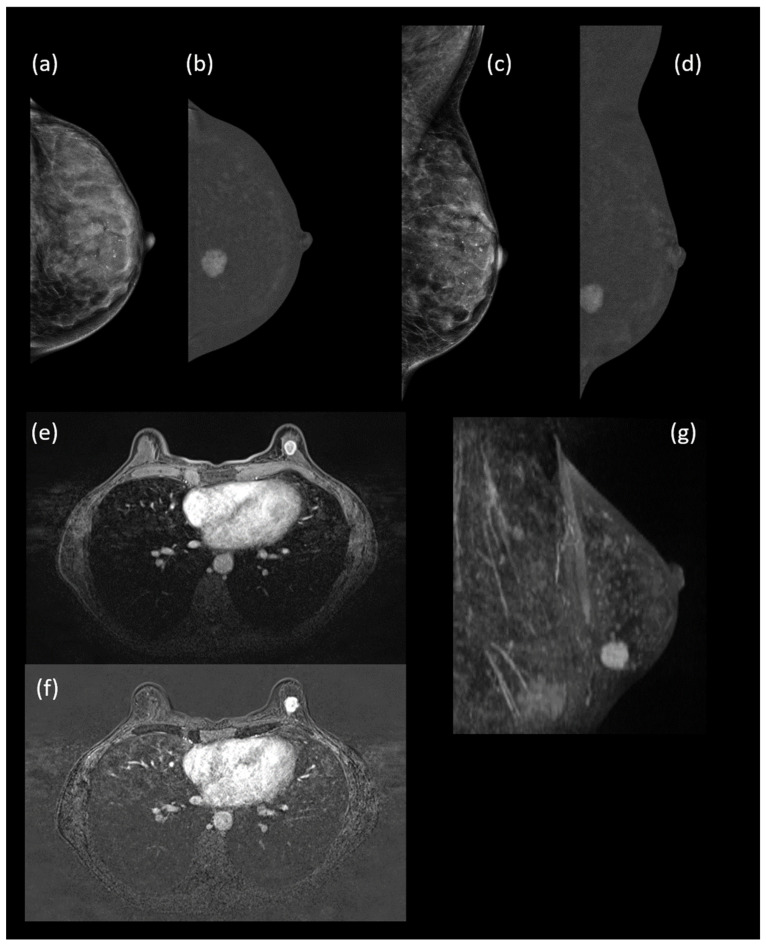
Imaging findings in a 54-year-old woman at intermediate risk for breast cancer (estimated lifetime risk 22.7%) with dense breasts. Low-energy contrast-enhanced mammography (LE-CEM) images in cranio-caudal (CC) and medio-lateral oblique (MLO) projections (**a**,**c**) show no clearly identifiable lesion, while the corresponding dual-energy subtraction (DES) CEM images (**b**,**d**) demonstrate a conspicuous enhancing mass in the left breast, highlighting the superior lesion detectability of CEM compared with LE-CEM. Breast MRI shows concordant findings, with the lesion clearly visible and suspicious on axial T1-weighted imaging (**e**), post-contrast subtraction images (**f**), and sagittal maximum intensity projection (MIP) reconstruction (**g**). Histopathological analysis confirmed an invasive ductal carcinoma, histological grade 2.

Figure 4 illustrates a representative case in which contrast enhancement enabled lesion detection on CEM despite negative low-energy images, with concordant MRI findings and histopathologic confirmation.

### 3.5. Tumor Characteristics and Detection

Table 5 summarizes tumor characteristics and the distribution of cancers missed by each modality.

Among the 119 cancers, invasive ductal carcinoma (IDC) predominated (82/119, 68.9%), followed by invasive lobular carcinoma (ILC, 26/119, 21.8%) and ductal carcinoma in situ (DCIS, 11/119, 9.2%). CEM missed one IDC (1.2%) and one ILC (3.8%), while MRI missed one IDC (1.2%), one ILC (3.8%), and two DCIS (18.2%). Only three tumors were missed by both modalities.

Most invasive tumors were grade 2 (48.1%) or grade 3 (39.8%), with few missed cases across all grades. Tumor receptor status and HER2 positivity showed no consistent pattern in missed cases, and tumor size did not appear to affect modality performance. Overall, missed cancers were few and distributed similarly between CEM and MRI, suggesting comparable detection across tumor characteristics (Table 5).

### 3.6. Radiation Dose

Mean glandular dose (MGD) per view for CEM increased progressively with breast thickness (Table 6), ranging from 1.56 ± 0.27 mGy for breasts 16–25 mm thick to 3.10 ± 0.21 mGy for breasts > 75 mm. MGDs remained below the corresponding EU reference limits for digital mammography and tomosynthesis in breasts thicker than 45 mm, whereas values for thinner breasts slightly exceeded these limits. The limits are defined for PMMA phantoms, and reported values correspond to equivalent compressed breast thickness.

## 4. Discussion

In this cohort of women at intermediate and high risk for breast cancer, CEM demonstrated diagnostic performance noninferior to breast MRI and significantly superior to LE-CEM, used as a surrogate for digital mammography. Using a multireader, multicase design, CEM achieved diagnostic accuracy comparable to MRI, with nearly identical mean AUCs (0.936 for CEM vs. 0.933 for MRI) and no significant differences in sensitivity or specificity (Table 4). Noninferiority testing confirmed AUC equivalence within the prespecified −0.05 noninferiority margin. These findings support CEM as a viable alternative to MRI for surveillance in women at increased risk, particularly when MRI is unavailable or contraindicated.

Our results are consistent with prior studies reporting comparable sensitivity between CEM and MRI, with some suggesting higher specificity for CEM [31,32,33,34,35]. The combined availability of high-resolution morphologic information and functional enhancement may explain this pattern, particularly through improved depiction of calcifications and more direct lesion–background correlation. In contrast, MRI’s higher sensitivity to benign proliferative changes may contribute to lower specificity in surveillance settings [25,31,32]. Importantly, performance was consistent across readers with varying experience, underscoring the robustness and reproducibility of CEM interpretation in clinical practice.

The incremental diagnostic value of contrast enhancement was confirmed by the comparison between LE-CEM and CEM. In the overall cohort, sensitivity increased from 68.9% with LE-CEM to 83.0% with CEM, with only a modest reduction in specificity (92.5% to 90.8%), resulting in a substantial increase in mean AUC. These findings align with prior reports demonstrating improved cancer detection with CEM compared with standard mammography, particularly in women with dense breasts [9,10,36]. The observed gains likely reflect the added functional information provided by iodinated contrast which enhances lesion conspicuity, especially for non-calcified or invasive tumors that may be occult on low-energy images [37,38].

In stratified analyses, overall performance patterns between CEM and MRI remained similar across risk, breast density, and menopausal subgroups. However, formal noninferiority was not demonstrated in the high-risk and post-menopausal strata, as confidence intervals marginally crossed the prespecified margin despite small absolute differences in AUC. These subgroup findings should be interpreted cautiously because the study was not specifically powered for noninferiority testing within strata. Importantly, the high-risk subgroup represented a substantial proportion of the cohort; therefore, these findings warrant further investigation rather than being attributed solely to sample size. Dedicated prospective studies, ideally randomized and adequately powered for subgroup evaluation, are warranted to clarify whether these observations reflect true performance differences or statistical variability.

Tumor-characteristic analysis showed low and comparable miss rates for CEM and MRI across histologic types, grades, receptor status, and tumor size, supporting the overall robustness of CEM performance.

From a practical standpoint, CEM offers several workflow advantages over MRI. Acquisition times for CEM are generally 5–10 min per patient for standard bilateral CC and MLO views, compared with 20–40 min for breast MRI, including positioning and dynamic contrast sequences [39]. CEM can be performed on widely available digital mammography units, reducing logistical complexity and allowing higher patient throughput. Interpretation times are also shorter, with most cases read within 5–7 min compared with 10–15 min for MRI, depending on sequences and postprocessing. Cost analyses suggest that CEM is less expensive than MRI, with examination costs reduced by more than 50% [40]. These factors support CEM as a practical and accessible alternative for high-risk surveillance. CEM contrast agents are generally safe, with low rates of allergic reactions, but caution is warranted in patients with severe iodine allergy or impaired renal function [41,42].

Additional considerations include background parenchymal enhancement (BPE), which can affect image quality, although MRI acquisition is typically synchronized with the patient’s menstrual cycle, whereas CEM is generally performed regardless of cycle timing [43,44]. Microcalcifications remain well visualized on LE-CEM images, highlighting the complementary nature of LE and dual-energy subtraction images. Radiation exposure, sequential image acquisition, and geometrical limitations near the chest wall or axilla should also be considered when planning surveillance protocols.

Mean glandular dose per view for CEM remained within clinically acceptable ranges across breast thickness categories, supporting its safe application in surveillance while delivering substantial diagnostic benefit [20,21]. Cumulative exposure, however, depends on the number of views and the interval of repeated examinations, which is particularly relevant for long-term follow-up in high-risk populations.

This study has limitations. Although imaging data were prospectively collected, the analysis was retrospective and conducted at a single institution, which may introduce selection bias. MRI and CEM were interpreted by different reader groups to minimize recall bias, precluding direct intra-reader comparisons. Verification bias cannot be fully excluded, as benign findings and negative cases were primarily confirmed by imaging follow-up rather than histopathology; however, this bias is mitigated by a minimum 24-month follow-up. Additionally, while standard diagnostic metrics such as sensitivity, specificity, and AUC are robust in this MRMC framework, other commonly reported screening indicators—such as cancer detection rate, false-negative rate, recall rate, biopsy rate, PPV1, and PPV2—cannot be reliably calculated in this enriched, retrospective cohort. Furthermore, PPV and NPV are prevalence-dependent and would not reflect performance in a routine screening population.

Despite these limitations, the large cohort, multireader design, standardized statistical framework, and rigorous follow-up strengthen the validity of our findings. Future research should include multicenter prospective trials, ideally randomized, to confirm these results in diverse populations, evaluate long-term outcomes, and determine how contrast-enhanced mammography can be optimally integrated into personalized, risk-based surveillance strategies.

## 5. Conclusions

CEM demonstrated diagnostic accuracy comparable to breast MRI and clearly superior to mammography in women at increased risk for breast cancer. These findings support the consideration of CEM as a practical, cost-effective, and widely accessible alternative to MRI for high-risk surveillance. Further multicenter prospective studies are warranted to confirm these results, particularly in high-risk subgroups, and to define the optimal integration of CEM into personalized, risk-adapted breast imaging strategies.

## Figures and Tables

**Table 1:** Study population characteristics.

Characteristic		Value
Age (mean ± SD)		49.8 ± 9.0 years
BMI (mean ± SD)		23.8 ± 4.4 kg/m^2^
Menopausal State	Premenopausal	242/461 (52.5%)
	Perimenopausal	46/461 (10.0%)
	Post-Menopausal	173/461 (37.5%)
Breast density *	a	25/461 (5.4%)
	b	68/461 (14.8%)
	c	141/461 (30.6%)
	d	227/461 (49.2%)
Breast cancer risk	Intermediate **	135/461 (29.3%)
Germline mutations (high-risk women only)	High ***	326/461 (70.7%)
	BRCA1	48/326 (14.7%)
	BRCA2	63/326 (19.3%)
	PALB2	2/326 (0.6%)
	No mutation	213/326 (65.3%)

SD, standard deviation. BMI, body mass index. * Breast density categories (BIRADS-like) were derived from volumetric breast density measured by Volpara software. ** Intermediate = women with lifetime risk for breast cancer between 17% and 30% (Tyrer–Cuzick risk model). *** High = women with lifetime risk above 30% (Tyrer–Cuzick risk model). BRCA1/2, breast cancer gene 1 and 2 mutations. PALB2, partner and localizer of BRCA2 gene mutation.

**Table 2:** Diagnostic performance of LE-CEM, CEM, and MRI per reader, with 95% confidence intervals, and mean values for each reader group.

Reader	Modality	Sensitivity (%) [95% CI]	Specificity (%) [95% CI]	AUC [95% CI]
R1	LE-CEM	73.9 [65.1, 81.6]	93.9 [90.8, 96.2]	0.897 [0.838, 0.920]
	CEM	89.1 [82.0, 94.1]	92.1 [88.7, 94.7]	0.953 [0.908, 0.967]
R2	LE-CEM	57.1 [47.7, 66.2]	97.7 [95.4, 99.0]	0.864 [0.772, 0.865]
	CEM	73.9 [65.1, 81.6]	95.3 [92.5, 97.3]	0.941 [0.882, 0.949]
R3	LE-CEM	68.9 [59.8, 77.1]	87.1 [83.1, 90.5]	0.821 [0.742, 0.840]
	CEM	84.9 [77.2, 90.8]	88.0 [84.1, 91.3]	0.928 [0.874, 0.943]
R4	LE-CEM	75.6 [66.9, 83.0]	91.5 [88.0, 94.2]	0.840 [0.803, 0.890]
	CEM	84.0 [76.2, 90.1]	87.7 [83.8, 91.0]	0.924 [0.862, 0.931]
R5	MRI	84.9 [77.2, 90.8]	91.8 [88.7, 94.7]	0.941 [0.886, 0.950]
R6	MRI	89.9 [83.0, 94.7]	78.9 [74.2, 83.1]	0.932 [0.871, 0.936]
R7	MRI	80.7 [72.4, 87.3]	92.1 [88.7, 94.7]	0.919 [0.872, 0.945]
R8	MRI	89.1 [82.0, 94.1]	83.3 [79.0, 87.1]	0.940 [0.893, 0.954]
Mean R1–R4	LE-CEM	68.9 [56.8, 81.1]	92.5 [85.9, 99.2]	0.856 [0.803, 0.909]
	CEM	83.0 [73.6, 92.4]	90.8 [95.5, 96.1]	0.936 [0.911, 0.962]
Mean R5–R8	MRI	86.1 [79.3, 93.0]	86.5 [76.7, 96.4]	0.933 [0.905, 0.960]

LE-CEM, low-energy contrast-enhanced mammography used as a surrogate of digital mammography. MRI, magnetic resonance imaging. AUC, area under the receiver operating characteristic curve.

**Table 3:** Mean diagnostic performance of LE-CEM, CEM, and MRI, with 95% confidence intervals, stratified by risk, breast density, and menopausal status.

Stratification	Subgroup	Modality	Sensitivity (%) [95% CI]	Specificity (%) [95% CI]	AUC [95% CI]
Risk	Intermediate (n = 135)	LE-CEM	77.7 [65.4, 89.9]	89.1 [81.7, 96.5]	0.880 [0.815, 0.944]
		CEM	89.8 [80.5, 99.0]	85.1 [77.8, 92.5]	0.956 [0.935, 0.976]
		MRI	92.0 [85.3, 98.8]	85.9 [75.3, 96.4]	0.957 [0.932, 0.980]
	High (n = 326)	LE-CEM	58.0 [44.2, 71.9]	93.4 [86.8, 99.9]	0.775 [0.646, 0.903]
		CEM	74.5 [62.6, 86.5]	92.2 [87.0, 97.4]	0.903 [0.857, 0.948]
		MRI	78.8 [68.6, 88.9]	86.7 [77.2, 96.3]	0.899 [0.851, 0.948]
Breast Density	Low (a + b) (n = 93)	LE-CEM	60.3 [37.4, 83.2]	96.4 [90.1, 100.0]	0.860 [0.743, 0.976]
		CEM	75.0 [55.9, 94.1]	93.8 [89.8, 97.7]	0.930 [0.882, 0.978]
		MRI	85.3 [68.5, 100.0]	89.8 [85.3, 94.3]	0.927 [0.867, 0.987]
	High (c + d) (n = 368)	LE-CEM	70.3 [59.2, 81.5]	91.4 [84.7, 98.2]	0.840 [0.762, 0.914]
		CEM	84.3 [76.0, 92.6]	89.9 [83.4, 96.5]	0.936 [0.910, 0.963]
		MRI	86.3 [79.8, 92.8]	85.6 [73.7, 97.6]	0.932 [0.902, 0.961]
Menopausal Status	Pre/Peri (n = 288)	LE-CEM	65.3 [54.2, 76.4]	91.1 [85.1, 97.1]	0.809 [0.725, 0.893]
		CEM	82.8 [74.7, 91.0]	88.9 [82.6, 95.2]	0.927 [0.892, 0.962]
		MRI	83.2 [74.0, 92.4]	83.3 [68.8, 97.8]	0.906 [0.859, 0.954]
	Post (n = 173)	LE-CEM	73.6 [58.6, 88.5]	95.2 [86.8, 100.0]	0.882 [0.798, 0.966]
		CEM	83.2 [69.1, 97.2]	94.2 [89.7, 98.7]	0.950 [0.910, 0.991]
		MRI	89.9 [83.0, 96.8]	92.6 [89.1, 96.0]	0.965 [0.936, 0.995]

**Table 4:** CEM-to-MRI differences for AUC, sensitivity, and specificity overall and across risk, breast density, and menopausal status subgroups.

CEM-to-MRI Difference			
Group/Subgroup	Metric	Mean Difference (95%CI)	*p*-Value
Overall (n = 461)	AUC	+0.004 (−0.034, 0.041)	0.839
	Sensitivity	−0.032 (−0.139, 0.076)	0.547
	Specificity	+0.042 (−0.050, 0.134)	0.318
Intermediate risk (n = 135)	AUC	−0.009 (−0.032, 0.030)	0.951
	Sensitivity	−0.023 (−0.126, 0.081)	0.648
	Specificity	−0.007 (−0.126, 0.111)	0.900
High Risk (n = 326)	AUC	+0.003 (−0.063, 0.069)	0.926
	Sensitivity	−0.042 (−0.196, 0.111)	0.582
	Specificity	−0.055 (−0.035, 0.145)	0.197
Low Density (n = 93)	AUC	+0.003 (−0.071, 0.077)	0.935
	Sensitivity	−0.103 (−0.348, 0.142)	0.399
	Specificity	+0.039 (−0.020, 0.099)	0.194
High Density (n = 368)	AUC	+0.004 (−0.035, 0.044)	0.826
	Sensitivity	−0.020 (−0.120, 0.081)	0.693
	Specificity	+0.043 (−0.068, 0.155)	0.395
Pre/Peri-Menopausal (n = 288)	AUC	+0.021 (−0.037, 0.079)	0.481
	Sensitivity	−0.004 (−0.124, 0.116)	0.950
	Specificity	+0.057 (−0.072, 0.186)	0.340
Post-Menopausal (n = 173)	AUC	−0.015 (−0.064, 0.035)	0.556
	Sensitivity	−0.067 (−0.212, 0.077)	0.344
	Specificity	+0.017 (−0.036, 0.069)	0.520

**Table 5:** Characteristics of tumors included in the study and distribution of cancers missed by each modality.

Tumor Characteristic	Total Number n (%)	Missed by CEM Only n (%)	Missed by MRI Only n (%)	Missed by Both n (%)
**Type**				
DCIS	11 (9.2%)	0 (0.0%)	2 (18.2%)	2 (18.2%)
IDC	82 (68.9%)	1 (1.2%)	1 (1.2%)	2 (2.4%)
ILC	26 (21.8%)	1 (3.8%)	1 (3.8%)	1 (3.8%)
Grade (invasive only) *				
1	13 (12.0%)	0 (0.0%)	1 (7.7%)	0 (0.0%)
2	52 (48.1%)	2 (3.8%)	0 (0.0%)	2 (3.8%)
3	43 (39.8%)	0 (0.0%)	0 (0.0%)	1 (2.3%)
**ER status (invasive only)**				
Positive	93 (86.1%)	2 (2.2%)	1 (1.1%)	2 (2.2%)
Negative	15 (13.9%)	0 (0.0%)	0 (0.0%)	1 (6.7%)
**PR status (invasive only)**				
Positive	78 (72.2%)	2 (2.6%)	1 (1.3%)	2 (2.6%)
Negative	30 (27.8%)	0 (0.0%)	0 (0.0%)	1 (3.3%)
**HER2 status (invasive only)**				
Positive	21 (19.4%)	0 (0.0%)	0 (0.0%)	0 (0.0%)
Negative	87(80.6%)	2 (2.3%)	1 (1.1%)	3 (3.4%)
**Tumor size**				
≤10 mm	38 (31.9%)	1 (2.6%)	1 (2.6%)	3 (7.9%)
11–20 mm	40 (33.6%)	1 (2.5%)	1 (2.5%)	1 (2.5%)
>20 mm	41 (34.5%)	0 (0.0%)	1 (2.4%)	1 (2.4%)

DCIS, ductal carcinoma in-situ. IDC, invasive ductal carcinoma. ILC, invasive lobular carcinoma. ER, estrogen receptor. PR, progesterone receptor. HER2, human epidermal growth factor receptor 2. * means applied only to invasive cancers.

**Table 6:** Mean glandular dose (MGD) per view for CEM across breast thickness intervals.

Breast Thickness Interval (mm)	N° of Patients	Mean CEM MGD per View (mGy)	EU Reference MGD Limits (Mammography and Tomosynthesis) (mGy)
16–25	16	1.6 ± 0.3	1.2 @20 mm
26–35	79	2.1 ± 0.2	1.5 @32 mm
36–45	83	2.2 ± 0.1	2.0 @45 mm
46–55	115	2.3 ± 0.2	2.5 @53 mm
56–65	68	2.5 ± 0.3	3.0 @60 mm
66–75	45	2.9 ± 0.3	4.5 @75 mm
>75	17	3.1 ± 0.2	6.5 @90 mm

EU reference limits represent the recommended maximum MGD per view from the European Guidelines for digital mammography and tomosynthesis. EU reference limits are defined for polymethyl methacrylate (PMMA) thicknesses. For each limit, the corresponding value shown represents the equivalent compressed breast thickness. CEM, contrast-enhanced mammography. MGD, mean glandular dose.

## Data Availability

The original data presented in the study are openly available in Zenodo at https://doi.org/10.5281/zenodo.18196716, accessed on 9 January 2026.

## Abbreviations

The following abbreviations are used in this manuscript:

CEM	Contrast-enhanced mammography
LE	Low-energy
HE	High-energy
MRI	Magnetic resonance imaging
MRMC	Multireader multicase
AUC	Area under the ROC curve
DES	Dual-energy subtraction
CC	Cranio-caudal
MLO	Medio-lateral oblique
MGD	Mean glandular dose

## Appendix A

### Appendix A.1

**Table A1:** Reader-specific confusion matrices listing true-positive (TP), false-positive (FP), true-negative (TN), and false-negative (FN) counts for low-energy CEM (used as a surrogate for digital mammography) and full contrast-enhanced mammography.

Reader	Treatment	True Positive (TPs)	False Positives (FPs)	True Negatives (TNs)	False Negatives (FNs)
1	LE-CEM	88	21	321	31
1	CEM	106	27	315	13
2	LE-CEM	68	8	334	51
2	CEM	88	16	326	31
3	LE-CEM	82	44	298	37
3	CEM	101	41	301	18
4	LE-CEM	90	29	313	29
4	CEM	100	42	300	19

### Appendix A.2

**Table A2:** Reader-specific confusion matrices listing true-positive (TP), false-positive (FP), true-negative (TN), and false-negative (FN) counts for CEM (contrast-enhanced mammography) and breast MRI (magnetic resonance imaging).

Reader	Treatment	True Positive (TPs)	False Positives (FPs)	True Negatives (TNs)	False Negatives (FNs)
1	LE-CEM	88	21	321	31
1	CEM	106	27	315	13
2	LE-CEM	68	8	334	51
2	CEM	88	16	326	31
3	LE-CEM	82	44	298	37
3	CEM	101	41	301	18
4	LE-CEM	90	29	313	29
4	CEM	100	42	300	19

### Appendix A.3

**Table A3:** CEM-to-LE-CEM differences for AUC, sensitivity, and specificity overall and across risk, breast density, and menopausal status subgroups.

CEM-to-LE-CEM Difference			
Group/Subgroup	Metric	Mean Difference (95%CI)	*p*-Value
Overall (n = 461)	AUC	+0.081 (0.045, 0.116)	<0.001
	Sensitivity	+0.141 (0.083, 0.199)	<0.001
	Specificity	−0.018 (−0.046, 0.011)	0.178
Intermediate risk (n = 135)	AUC	+0.076 (0.020, 0.131)	<0.001
	Sensitivity	+0.121 (0.074, 0.169)	<0.001
	Specificity	−0.040 (−0.088, 0.009)	0.099
High Risk (n = 326)	AUC	+0.128 (0.017, 0.238)	0.029
	Sensitivity	+0.165 (0.072, 0.259)	0.002
	Specificity	−0.012 (−0.039, 0.015)	0.316
Low Density (n = 93)	AUC	+0.070 (−0.026, 0.167)	0.148
	Sensitivity	+0.147 (0.035, 0.259)	0.010
	Specificity	−0.026 (−0.073, 0.021)	0.229
High Density (n = 368)	AUC	+0.096 (0.030, 0.162)	0.011
	Sensitivity	+0.140 (0.078, 0.202)	<0.001
	Specificity	−0.015 (−0.044, 0.014)	0.252
Pre/Peri-Menopausal (n = 288)	AUC	+0.118 (0.045, 0.191)	0.006
	Sensitivity	+0.175 (0.098, 0.253)	<0.001
	Specificity	−0.021 (−0.052, 0.009)	0.137
Post-Menopausal (n = 173)	AUC	+0.069 (−0.007, 0.144)	0.068
	Sensitivity	+0.096 (0.041, 0.152)	0.001
	Specificity	−0.010 (−0.045, 0.024)	0.509
